# Micellar Nanocarriers of Hydroxytyrosol Are Protective against Parkinson’s Related Oxidative Stress in an In Vitro hCMEC/D3-SH-SY5Y Co-Culture System

**DOI:** 10.3390/antiox10060887

**Published:** 2021-05-31

**Authors:** Leah Mursaleen, Brendon Noble, Satyanarayana Somavarapu, Mohammed Gulrez Zariwala

**Affiliations:** 1 Centre for Nutraceuticals, School of Life Sciences, University of Westminster, 115 New Cavendish Street, London W1W 6UW, UK; w1655446@my.westminster.ac.uk (L.M.); b.noble@westminster.ac.uk (B.N.); 2 Department of Pharmaceutics, UCL School of Pharmacy, 29–39 Brunswick Square, London WC1N 1AX, UK; 3 Cure Parkinson’s, 120 New Cavendish Street, Fitzrovia, London W1W 6XX, UK

**Keywords:** hydroxytyrosol, deferoxamine, Parkinson’s disease, oxidative stress, antioxidant, neurodegeneration, blood–brain barrier, Transwell^®^, Pluronic^®^ F68, dequalinium, micelles

## Abstract

Hydroxytyrosol (HT) is a natural phenolic antioxidant which has neuroprotective effects in models of Parkinson’s disease (PD). Due to issues such as rapid metabolism, HT is unlikely to reach the brain at therapeutic concentrations required for a clinical effect. We have previously developed micellar nanocarriers from Pluronic F68^®^ (P68) and dequalinium (DQA) which have suitable characteristics for brain delivery of antioxidants and iron chelators. The aim of this study was to utilise the P68 + DQA nanocarriers for HT alone, or in combination with the iron chelator deferoxamine (DFO), and assess their physical characteristics and ability to pass the blood–brain barrier and protect against rotenone in a cellular hCMEC/D3-SH-SY5Y co-culture system. Both HT and HT + DFO formulations were less than 170 nm in size and demonstrated high encapsulation efficiencies (up to 97%). P68 + DQA nanoformulation enhanced the mean blood–brain barrier (BBB) passage of HT by 50% (*p* < 0.0001, *n* = 6). This resulted in increased protection against rotenone induced cytotoxicity and oxidative stress by up to 12% and 9%, respectively, compared to the corresponding free drug treatments (*p* < 0.01, *n* = 6). This study demonstrates for the first time the incorporation of HT and HT + DFO into P68 + DQA nanocarriers and successful delivery of these nanocarriers across a BBB model to protect against PD-related oxidative stress. These nanocarriers warrant further investigation to evaluate whether this enhanced neuroprotection is exhibited in in vivo PD models.

## 1. Introduction

Hydroxytyrosol (HT) is a natural phenolic compound that has generated interest in Parkinson’s disease (PD) research due to its antioxidant properties. HT is a major component of olive oil and therefore prominent in the Mediterranean diet [1,2], which has been related to lower mortality [3,4], improved cardiovascular health [5,6] and slower cognitive decline [7]. A wide body of research supports a protective role of HT against neurodegeneration [8,9,10,11,12,13].

HT’s catecholic structure provides reactive oxygen species (ROS) scavenging properties through the ability of the benzene ring-bound hydroxyl groups to donate either an electron or hydrogen atom to stabilise ROS [14,15,16].

In PD-related cellular models, HT protects dopaminergic neurons against cell death following oxidative stress [8,11] and protects against alpha synuclein fibril formation and aggregation in the PC12 cell line [17]. Animal studies have shown resistance to oxidative stress via reduced lipid peroxidation in dissociated brain cells following administration of 100 mg/kg HT for 12 days [18], suggesting that HT can access the brain. Although other studies have also shown that HT can cross the blood–brain barrier (BBB), brain uptake of HT is lower than uptake in other organs and only reaches the brain in micromolar concentrations [11,19]. For example, D’Angelo et al. [19] showed that rat brain tissue resulted in only 0.31% of the 1.5 mg/kg administered dose, 5 min after intravenous injection. Additionally, Wu et al. [11] showed the maximum concentration reaching rat brain was 2.1 μg/mL 15 min after an intravenous dose of 100 mg/kg. Furthermore, HT is rapidly metabolised and is therefore unlikely to reach the brain at therapeutic concentrations required for a clinical effect [20].

Nanocarriers can enhance the potency, stability, bioavailability, and the passage across biological membranes of incorporated compounds [21,22,23,24]. Pluronic F68 (P68) + dequalinium (DQA) nanocarriers have been successfully used to deliver the antioxidants curcumin and *N*-acetylcysteine, alone and in combination with the iron chelator deferoxamine (DFO), to mitochondria within SH-SY5Y cells to protect against a rotenone model of PD [24,25]. These nanocarriers exhibit many characteristics that make them suitable for brain penetrance, for example small particle size (<200 nm) and relatively neutral charge (<10 mV) [23,26,27,28,29,30,31,32,33]. They also target the mitochondria, the main site of iron-induced oxidative stress and the associated dopaminergic cell death evident in PD [24,25,34,35,36,37,38,39,40,41,42,43]. The combination of antioxidants and iron chelators for the treatment of PD may provide a promising strategy to limit the degenerative process in PD due to the dual approach of free radical scavenging and iron chelation to limit detrimental free iron availability. Therefore, the aim of this study was to utilise the P68 + DQA nanocarriers for HT alone, or in combination with DFO, and assess their physiochemical characteristics and ability to pass the BBB and protect against rotenone in a cellular hCMEC/D3-SH-SY5Y co-culture system.

## 2. Materials and Methods

All chemicals were analytical grade or cell culture grade where applicable (unless otherwise stated). The SH-SY5Y cell line (CRL-2266) was purchased from the American Type Culture Collection (ATCC, Manassas, VA, USA). The hCMEC/D3 cell line was gifted from Simon McArthur (Queen Mary University of London, London, UK), previously purchased from VHBio Ltd. (Gateshead, UK).

Collagen from calf skin, Dulbecco’s phosphate buffered saline (DPBS), EGM-2 MV Microvascular Endothelial Cell Growth Medium-2 BulletKit^TM^, fibronectin (bovine plasma), Hank’s Buffered Saline Solution (HBSS) and HEPES Buffer (1 M) were purchased from Lonza (Slough, UK). The 100× antibiotic-antimycotic, Dulbecco’s modified Eagle medium (DMEM) Glutamax^®^, foetal bovine serum (FBS), L-glutamine, methanol (HPLC grade), Minimum Essential Media (MEM) and poloxomer 68 (Pluronic^®^ F68) were supplied by Fisher Scientific, Loughborough, UK. Dequalinium chloride hydrate (95%), 3-Hydroxytyrosol (≥98%), rotenone (≥95%), Thiazolyl Blue Tetrazolium Blue (MTT), dimethyl sulfoxide (DMSO), and 2, 4, 6-tripyridyl-s- triazine and iron(III) Chloride Hexahydrate were purchased from Sigma-Aldrich, Gillingham, UK. The mitochondrial hydroxyl radical detection assay kit (cat no. ab219931) was purchased from Abcam, Cambridge, UK. Milli-Q water (water purified through a 0.22 μm membrane filter with a resistivity of 18.2 MΩ) was used in the preparation of experimental reagents. Millex-MP sterile filters were purchased from Millipore (Watford, UK). Nunc (Roskilde, Denmark) supplied the culture flasks and all other plastics were purchased from Corning (Flintshire, UK).

### 2.1. Preparation of HT and HT + DFO Micellar Nanoformulations

All nanoformulations were prepared using a modified thin-film hydration method [22,24,25,44]. Briefly, 10 mL of methanol was used to dissolve P68 and DQA with HT or HT + DFO at certain ratios (Table 1). Using the Hei-VAP Advantage Rotary Evaporator (Heidolph, Schwabach, Germany), evaporation of the methanol was carried out in a vacuum (200 rpm, 80 °C) to obtain a thin film of drug-loaded nanocarriers. Then, 10 mL of distilled water was then used to hydrate the resultant film. This was mixed thoroughly at 80 °C for 1–2 min and sonicated for another 1 min using a VWR Ultrasonic cleaner bath USC300T (VWR International Limited, Lutterworth, UK) to fully dissolve the film in the water. The obtained solution was filtered through a sterile 0.22 μm filter to remove any unloaded HT and DFO. Some samples were freeze dried (lyophilized) using a Virtis AdVantage 2.0 BenchTop freezedryer (SP Industries, Ipswich, UK) for the X-ray diffraction (XRD) and fourier-transform infrared (FTIR) analyses.

### 2.2. Size and Surface Charge of the Nanoformulations

The Zetasizer Nano ZS (Malvern Instruments, Worcestershire, UK) was used to analyze the dimensions and surface charge of the nanoformulations. Photon correlation spectroscopy was used to measure size distribution as Z-Ave hydrodynamic diameter and polydispersity index.

### 2.3. Determination of Drug Loading and Encapsulation Efficiency

Drug loading and encapsulation efficiency of the nanoformulations was studied using UV-Visible (UV-Vis) spectroscopy based on the calibration curves of the free drugs. Methanol and water were used in a 1:1 ratio to dissolve the carrier and release the drug to achieve a theoretical concentration of 20 μg/mL HT and DFO. HT and DFO content were calculated using UV-Vis spectroscopy (Cary 100 UV-Vis, Agilent Technologies, Santa Clara, CA, USA) at 280 and 204 nm, respectively. The following equations were used to calculate the percentage of drug loading and encapsulation efficiency:

Drug loading (%) = (determined mass of drug within nanocarriers/mass of drug-loaded nanocarriers) × 100(1)

Encapsulation efficiency (%) = (determined mass of drug within nanocarriers/theoretical mass of drug within nanocarriers) × 100(2)

### 2.4. Structural Analysis

The Rigaku MiniFlex600 x-ray diffractometer (Rigaku Corporation, Tokyo, Japan) was used for atomic and molecular structural analysis of the samples at a 5−35° range and step size of 0.01° (the scanning rate was 2°/min). XRD patterns were obtained for pure HT, DFO, P68 and DQA, as well as the P68 + DQA HT and HT + DFO nanocarriers (in lyophilized form). All XRD analysis was carried out at room temperature.

A PerkinElmer Spectrum 100 FTIR spectrometer (PerkinElmer, Waltham, MA, USA) was used to analyse the chemical structure of the pure drugs, nanocarriers alone, their physical mixtures and lyophilised drug-loaded nanocarriers from 650 to 4000 cm^−1^, at a resolution of 4 cm^−1^.

### 2.5. Antioxidant Power of the Antioxidant Nanoformulations

The modified ferric iron reducing antioxidant power (FRAP) assay was used to determine the potential antioxidant activity of HT-loaded nanoparticles compared to free HT at a range of concentrations, as previously described [22,25]. Briefly, FRAP reagent (a mixture of pH 3.6 acetate buffer, tripyridyl triazine, and iron (III) chloride) and samples of free and P68+DQA HT were incubated for 30 min at room temperature before being read at 593 nm. In line with previous reports [25,45,46], trolox was used as the standard and the antioxidant capacity of the samples was given as the trolox equivalent concentration.

### 2.6. SH-SY5Y Cell Culture and Cytotoxicity Testing of the Nanoformulations

SH-SY5Y cells were grown in plastic T75 (75 cm^2^) flasks at 37 °C (5% CO_2_) until 70% confluent. The culture media consisted of pH 7.4 DMEM-Glutamax^®^, 10% FBS and 1% antibiotic/antimycotic. Following trypsinisation, SH-SY5Y cells were seeded (1,000,000 cells/cm^2^) into 96-well plates ready for the experimental assays to be carried out.

For cytotoxicity evaluation of the drug-loaded nanoformulations, SH-SY5Y cells were treated with free HT and HT + DFO or the corresponding concentrations of each drug-loaded nanoformulation for 24, 48 and 72 h. The MTT assay was used to assess cell viability based on the reduction of the yellow thiazolyl blue tetrazolium bromide salts (3-(4, 5-dimethylthiazol-2-yl)-2, 5-diphenyltetrazolium bromide, MTT) to the purple formazan by mitochondrial dehydrogenases, as previously described [24,25]. Briefly, once confluent cells were treated with either free or nanoformulated HT at varying concentrations for up to 72 h. 20 μL of MTT diluted in DPBS (5 mg/mL) was then added to the cells. Following a 4 h incubation at 37 °C and aspiration of the wells, 100 μL of DMSO was used to dissolve any resulting formazan crystals and the plates were incubated for 15 min on a shaker (75 rpm). The absorbance was then read at 570 nm on a spectrophotometer.

### 2.7. hCMEC/D3 Cell Culture

An in vitro model of the BBB was created using the hCMEC/D3 cerebral microvascular endothelial cell line, as previously described [47,48,49,50,51,52]. hCMEC/D3 cells were grown in T75 flasks in a 5% CO_2_ environment at 37 °C in Microvascular Endothelial Cell Growth Medium-2 BulletKit^TM^ (EGM-2 MV) which is supplemented with 5% FBS, 0.04% hydrocortisone, 0.4% hFGF-B (human basic fibroblast growth factor), 0.1% VEGF (vascular endothelial growth factor), 0.1% R3-IGF-1 (human recombinant insulin-like growth factor), 0.1% ascorbic acid and 0.1% GA (gentamicin sulfate-amphotericin). Once sufficiently confluent (~70%), adherent cells were detached from the surface of the flasks using trypsin and the cells were seeded at 300,000 cells/cm^2^ into the 3.0 μm pore polycarbonate membrane inserts of 6- and 96-well Costar Transwell^®^ plates precoated with 1:20 type 1 collagen from calf skin: DPBS (1 h) and 1:100 fibronectin from bovine plasma: DPBS (1 h). The hCMEC/D3 cells were exposed to VEGF-free media for 72 h prior to testing to promote tight junction formation [48,50,52,53].

### 2.8. Trans-Endothelial Electrical Resistance Assessment

The resistance of the BBB model was assessed using trans-endothelial electrical resistance (TEER) measurements as previously described by Burkhart et al. [54]. TEER values were read using an epithelial Volt-Ohm meter and sterile Chopstick Electrodes and expressed as Ω·cm^2^ (resistance of the tissue (Ω) × membrane area (cm^2^)). Tight junctions increase the resistance, therefore, high TEER values are desired [51]. TEER values of hCMEC/D3 cells have been shown to reach 300 Ω·cm^2^ in the presence of hydrocortisone [49,53,55]). Therefore, before carrying out any of the BBB passage experiments, TEER values were measured each day post seeding into Transwell^®^ plates, until a resistance of close to 300 Ω·cm^2^ was reached.

### 2.9. Assessment of Nanocarrier Passage across the hCMEC/D3 BBB Model

The ability of the nanoformulations to pass across the model BBB was assessed using a transport assay as previously described [56,57]. Phenol red-free HBSS was used to carefully wash each chamber three times, avoiding disturbance to the hCMEC/D3 monolayer. In total, 1 and 2.5 mL HBSS was then added to the apical and basolateral chambers (respectively) and incubated for 10 min at 37 °C. The apical chamber was then aspirated and treated with 1.5 mL nanoformulated or corresponding free HT and HT + DFO treatments (in HBSS) at a range of concentrations for 1 h at 37 °C. Following sampling of the basolateral chambers, HT content was calculated using UV-Vis spectroscopy at 280 nm as described above. To assess the stability of the BBB model and potential cytotoxicity of the treatments, TEER measurements were taken immediately after each transport assay.

### 2.10. hCMEC/D3 and SH-SY5Y Co-Culture in the Costar Transwell^®^ System

The hCMEC/D3 cells were seeded at 300,000 cells/cm^2^ into the 3.0 μm pore polycarbonate membrane inserts of 96-well Costar Transwell^®^ plates as described above. In parallel, SH-SY5Y cells were seeded at 1,000,000 cells/cm^2^ into 96-well plates. Once the hCMEC/D3 cells reached a membrane potential of at least 300 Ω·cm^2^ and the SH-SY5Y cells reached confluence, the hCMEC/D3 cultured Transwell^®^ inserts were place into the 96-well plates containing the confluent SH-SY5Y cells ready for immediate treatment.

### 2.11. Assessment of the Protective Effects of the Nanocarriers against Rotenone Following Passage across the BBB Model

SH-SY5Y cells in the basolateral chamber of the Transwell^®^ co-culture system were treated with 200 μL MEM. hCMEC/D3 cells in the apical chamber were treated with 150 μL of nanoformulated or free HT or HT + DFO treatments (in HBSS) at a range of concentrations. The cells were then incubated at 37 °C for 1 h. The Transwell^®^ inserts containing the hCMEC/D3 cells were then removed and the SH-SY5Y cells were incubated for a further 2 h at 37 °C. Following incubation, SH-SY5Y cells were treated with 100 μM rotenone for 24 h at 37 °C. The MTT assay was then carried out as described above to assess the ability of the treatments to protect against reduced cell viability induced by rotenone after passing the BBB model.

The mitochondrial hydroxyl radical detection assay was conducted to assess the protective effects of the nanoformulations against rotenone induced oxidative stress in this co-culture model but using black-walled, clear-bottom 96-well microplates for the SH-SY5Y cells. This assay was carried out as previously described by Mursaleen et al. [25], in accordance with the manufacturer’s protocol (ab219931; Abcam, Cambridge, UK). Following treatment with free or nanoformulated HT and HT + DFO and the removal of the hCMEC/D3 cultured Transwell^®^ inserts (as described above), SH-SY5Y cells were washed with DPBS and treated for 1 h at 37 °C with 100 µL of 6.25X OH580 probe. The cells were then incubated with 100 µM rotenone for 24 h at 37 °C. DPBS was used to wash the cells before the fluorescence was read on the Fluostar Optima Fluorescence Plate Reader (BMG LABTECH, Aylesbury, UK).

### 2.12. Statistical Analysis

The mean of six replicates was calculated for each treatment in all experiments. Data are expressed as mean ± standard deviation (S.D.). Two-way analysis of variance (ANOVA) followed by the Tukey’s or Šidák multiple comparisons post hoc test was used to analyse the FRAP, TEER and BBB passage data. The MTT and mitochondrial hydroxyl assay results were analysed using one-way ANOVA followed by the Dunnett’s T3 post hoc test (PRISM software package, Version 8, Graphpad Software Inc., San Diego, CA, USA).

## 3. Results

Both HT and HT + DFO loaded nanocarriers exhibited high mean encapsulation efficiency (95% and 97%, respectively) (Table 1). The drug-loaded nanocarriers exhibited a significantly higher mean particle size compared to the unloaded blank nanoformulation (*p* < 0.0001) (Table 1). The addition of DFO into the formulation increased the mean encapsulation efficiency of HT by 2%. The mean size of the HT and HT + DFO loaded nanocarriers were 166 and 146 nm, respectively (Table 1). Although the addition of DFO to the HT P68 + DQA nanoformulation appeared to lower the particle size, this was not a significant difference (Table 1). The mean polydispersity indices of the nanoformulations were < 0.24 which indicates that the majority of the nanocarriers within each formulation sample were of similar size (Table 1). The mean surface charges of the drug loaded nanocarriers were moderately positive (7–10 mV) whereas the surface charge of the blank unloaded nanoformulation was slightly negative (−0.78 mV) (Table 1).

**Figure 1:**
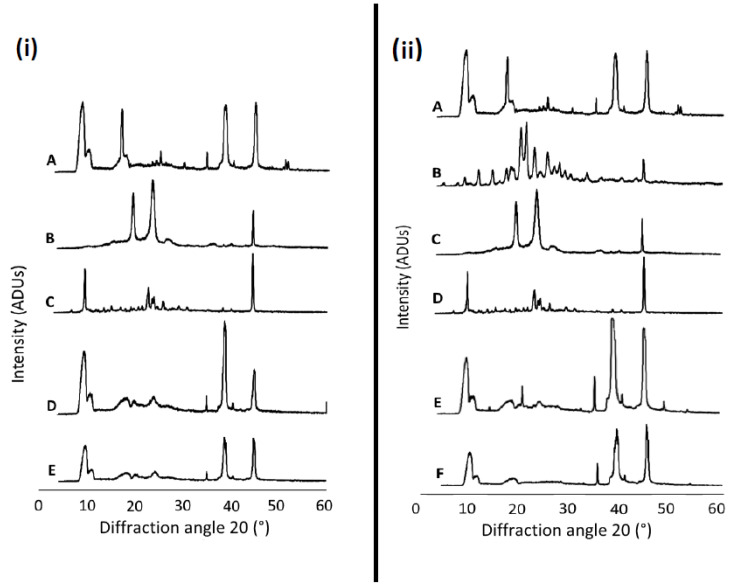
(**i**) P68+DQA HT. XRD spectra for (**A**) HT, (**B**) P68, (**C**) DQA, (**D**) P68, DQA and HT in a physical mixture (using the same ratio as the nanoformulation), (**E**) P68+DQA HT (lyophilized nanoformulation). (**ii**) P68+DQA HT+DFO. XRD spectra for (**A**) HT, (**B**) DFO, (**C**) P68, (**D**) DQA, (**E**) P68, DQA and HT in a physical mixture (using the same ratio as the nanoformulation), (**F**) P68+DQA HT+DFO (lyophilized nanoformulation). The P68, DQA, and DFO spectra are as previously reported by Mursaleen et al. [25]).

XRD spectra for free and nanoformulated HT and HT + DFO are shown in Figure 1 along with the physical mixture of the components of each nanoformulation and the individual formulation components (P68 and DQA). A high level of crystallinity is shown with the HT spectrum, with the main peaks at 8.74°, 10.2°, 16.98°, 25.02°, 29.98°, 34.66°, 38.3°, 44.9°, 51.44°, 51.58° (Figure 1(iA,iiA)). As previously reported by Mursaleen et al. [25], DFO has a large number of peaks indicating crystallinity (Figure 1(iiB)), whereas both P68 and DQA have less peaks within their spectra (Figure 1(iB,C,iiC,D)). Both the P68+DQA HT and HT + DFO lyophilized formulations present a more amorphous nature, with only 4 clear peaks (8.9, 34.7, 38.3 and 44.6°) (Figure 1(iE,iiF)). This reduction of peaks was not as prominent for the physical mixtures (Figure 1(iD,iiE)).

**Figure 2:**
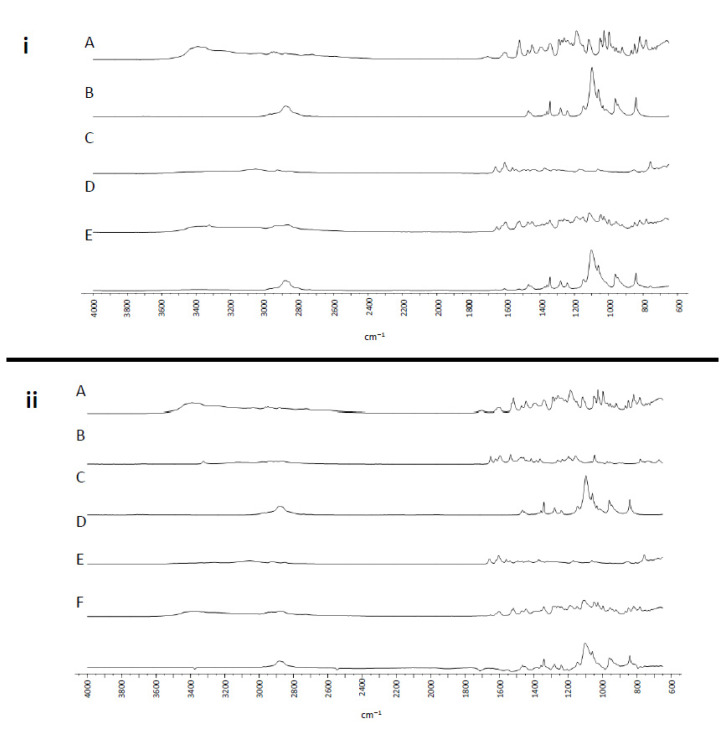
(**i**) P68 + DQA HT. FTIR spectra for (**A**) HT, (**B**) P68, (**C**) DQA, (**D**) a physical mixture of P68, DQA and HT in the same ratio as the nanoformulation and (**E**) lyophilized P68 + DQA HT nanoformulation. (**ii**) P68 + DQA HT + DFO. FTIR spectra for (**A**) HT, (**B**) DFO, (**C**) P68, (**D**) DQA, (**E**) a physical mixture of P68, DQA, HT and DFO in the same ratio as the nanoformulation and (**F**) lyophilized P68 + DQA HT + DFO nanoformulation.

Figure 2 shows the FTIR spectrum for the P68 + DQA HT and HT + DFO lyophilized formulations, the individual components of each formulation and the physical mixture of these components. The HT FTIR spectrum (Figure 2(iA,iiA)) indicates the presence of characteristic hydroxyl groups with sharp peaks at 3385, 1447 and 1187 cm^−1^.

The strong peak at 1606 cm^−1^ represents the stretching vibrations of the C=C bonds within the aromatic ring. The FTIR spectrum for DFO (Figure 2(iiB)) shows peaks representing stretching vibrations of characteristic bonds for OH (3324, 1472, 1386 cm^−1^), C=O (2864, 1652, 1536, 974 and 897 cm^−1^), NH_2_ (1598 cm^−1^), and N-H (3115, and 1608 cm^−1^). The FTIR spectrum for P68 (Figure 2(iC,iiD)) shows peaks at around 2880, 1060 and 841 cm^−1^ representing the vibrations of the hydroxyl groups and the stretching vibrations of the symmetrical C-O and asymmetrical C-O of the ether groups, respectively. The sharp peak at 1279 cm^−1^ represents the vibrations of the methylene group. The DQA spectrum (Figure 2(iC,iiD)) shows the absorption peaks at 3342 and 3264 cm^−1^ which represent the two primary amine groups for the asymmetric and symmetric N-H stretches. The sharp peak at 1605 cm^−1^ represents the vibration of aromatic C=C bonds. Methyl group stretching and deformation is represented by the peaks at 2928 and 2847 cm^−1^. The physical mixtures for each formulation (Figure 2(iD,iiE)) show peaks corresponding to each constituent within the mixture. However, the HT and DFO peaks appear less intense in the mixtures (Figure 2). The FTIR spectrum for each of the lyophilized formulations are similar to those of the physical mixtures but in each case the peaks corresponding to the HT and DFO elements are less intense (Figure 2(iD,iE,iiE,iiF)).

**Figure 3:**
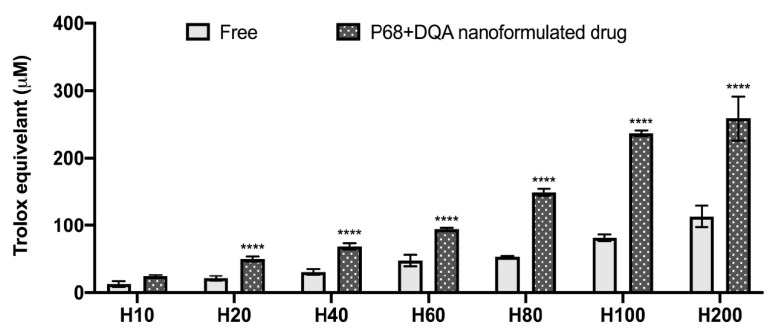
Antioxidant activity of free and P68 + DQA nanoformulated 10–200 μM HT measured by the FRAP assay (mean ± S.D., *n* = 6). * represents significance values of nanoformulated drug compared to free drug within the same treatment condition (**** *p* < 0.0001).

Figure 3 shows the antioxidant capacity of free and P68 + DQA nanoformulated HT (10–200 μM), analysed using the FRAP assay. When comparing the different concentrations of HT (F(6, 70) = 427.5, *p* < 0.0001) and free vs nanoformulated HT (F(1, 70) = 1029, *p* < 0.0001), significant differences were observed. Each P68 + DQA concentration of HT, except 10 μM, exhibited significantly higher trolox equivalent antioxidant capacity than the corresponding concentrations of free HT (*p* < 0.0001) (Figure 3). The percentage increase in antioxidant capacity of the P68 + DQA HT compared to the free HT preparations were over 100% for most concentrations (20, 40, 80, 100, and 200 μM) but all were over 93% (Figure 3).

**Figure 4:**
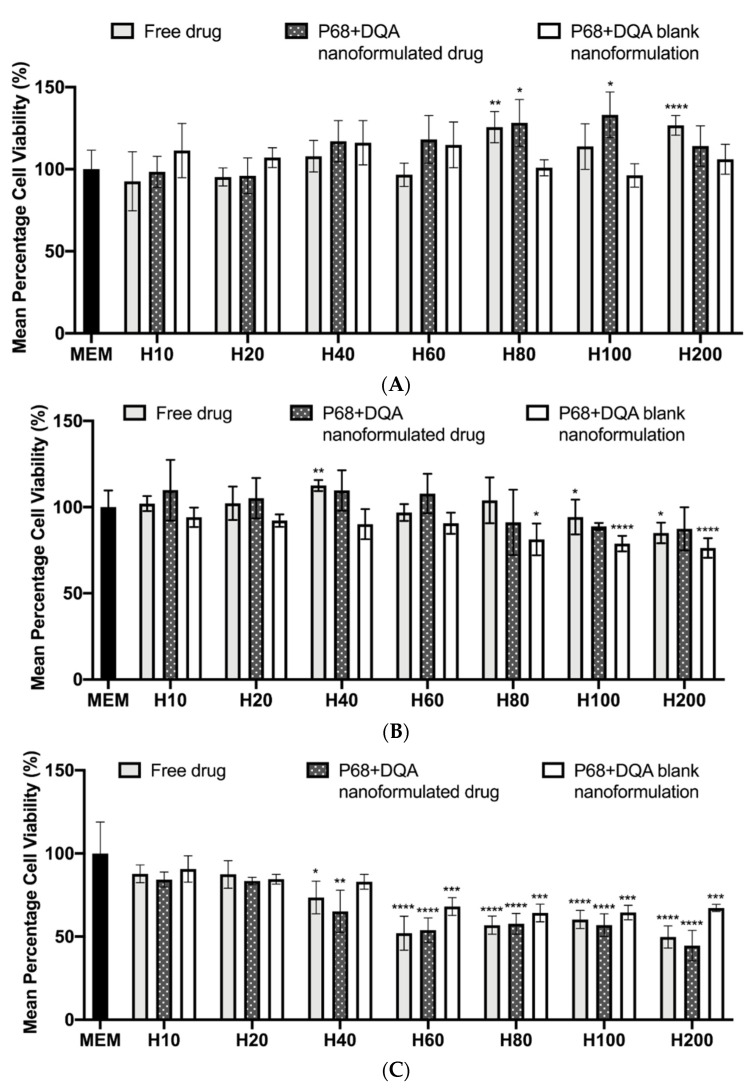
(**A**) MTT assay results of 24 h 10–200 μM HT treatment. MEM represents the control condition, cells only treated with media (mean ± S.D., *n* = 6). (**B**) Corresponding MTT assay results for 48 h HT treatments. (**C**) Corresponding MTT assay results for 72 h HT treatments. * represents significance values of the treatment conditions compared to the control condition (**** *p* < 0.0001, *** *p* < 0.001, ** *p* < 0.01, * *p* < 0.05).

The same concentration ranges of free and P68 + DQA HT were then tested on the SH-SY5Y cell line to evaluate the cytotoxicity of each concentration, using the MTT assay. Cell viability was maintained at control levels or above following treatment for 24 h with 10–200 μM free and P68 + DQA HT (F(21, 81.45) = 6.801, *p* < 0.0001) (Figure 4). No significant reduction in cell viability was observed for any concentration of HT (free or formulated) following 48 h treatment (Figure 4B). Although Figure 4B shows a significant reduction of cell viability compared to control, when treating with 200 μM free HT (*p* = 0.0135) and the corresponding blank formulations at 80 μM (*p* = 0.0338) and 100–200 μM (*p* < 0.0001), cell viability was above 80% in all cases (F(21, 57.30) = 6.9155, *p* < 0.0001). By the 72 h time point, a significant reduction in cell viability was observed for free HT at 40 μM (*p* = 0.0141), free and P68 + DQA formulated HT at 60–200 μM (*p* < 0.0001), and with the corresponding blank formulations (*p* < 0.0005) (Figure 4C). However, no cytotoxicity was observed with 40 μM treatment of free and P68 + DQA formulated HT (F(21, 73.11) = 29.41, *p* < 0.0001) (Figure 4C).

The 10 and 20 μM concentrations of free and P68 + DQA HT exhibited no cytotoxicity at any time point (24, 48 or 72 h) and were therefore used in the subsequent evaluations. The 50 and 100 μM DFO were used for the combined treatments based on our previous reports [24,25].

**Figure 5:**
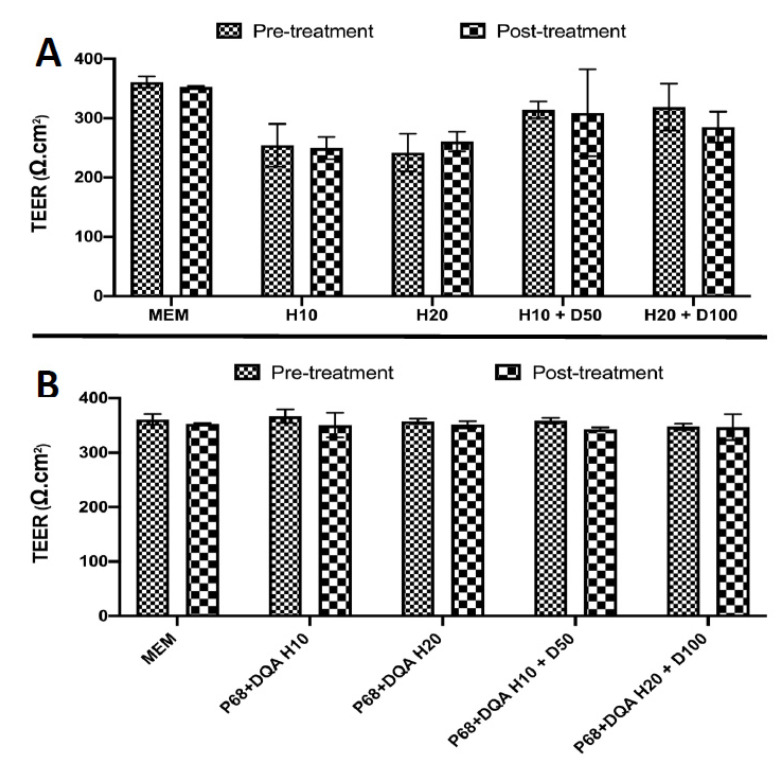
(**A**) Mean TEER of hCMEC/D3 cell monolayers on day 5 post seeding before (pre-treatment) and after (post-treatment) treatment with HT and combined HT and DFO free drug treatments. (**B**) Corresponding TEER results for the P68 + DQA nanoformulated HT and HT + DFO treatments.

The mean TEER of hCMEC/D3 cell monolayers grown on Transwell^®^ inserts peaked at 320 Ω·cm^2^ on day five post seeding and no significant difference in TEER was observed following any of the free and nanoformulated HT and HT + DFO treatments (Figure 5).

**Figure 6:**
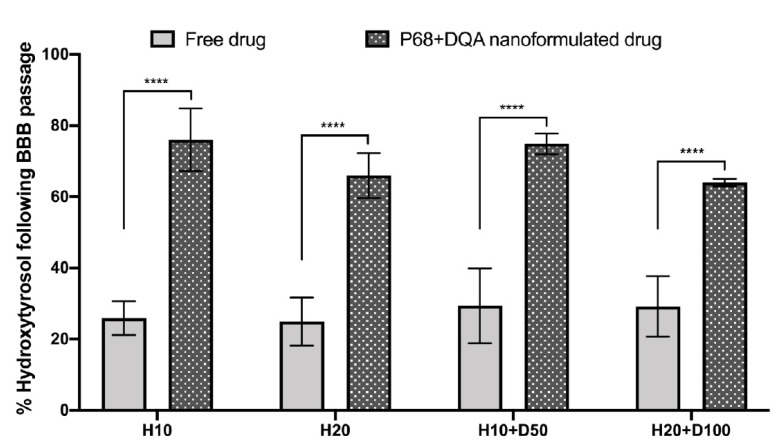
Mean percentage of HT in the basolateral compartment of the hCMEC/D3 Transwell^®^ system following 60 min treatment with free or P68 + DQA HT (10 and 20 µM) and combined HT and DFO (10 and 20 µM HT + 50 and 100 µM DFO, respectively). Percentage HT = ((absorbance of the basolateral compartment sample − control)/(absorbance of the treatment- control)) × 100, where the absorbance was read at 280 nm and the control was MEM. * represents significance values of nanoformulated drug compared to free drug within the same treatment condition (**** *p* < 0.0001).

When comparing the P68 + DQA nanoformulation and free drug treatments of HT and HT + DFO, significant differences in the percentage of HT were observed following BBB passage (F(1, 32) = 406.4, *p* < 0.0001) (Figure 6). All P68 + DQA formulations of HT and HT + DFO resulted in significantly more HT (between 34.8 and 50.1%) compared to the free drug treatments (*p* < 0.0001 in all cases), reaching more than 76% HT following passage across the hCMEC/D3 monolayer with the P68 + DQA 10 µM HT treatment (Figure 6).

**Figure 7:**
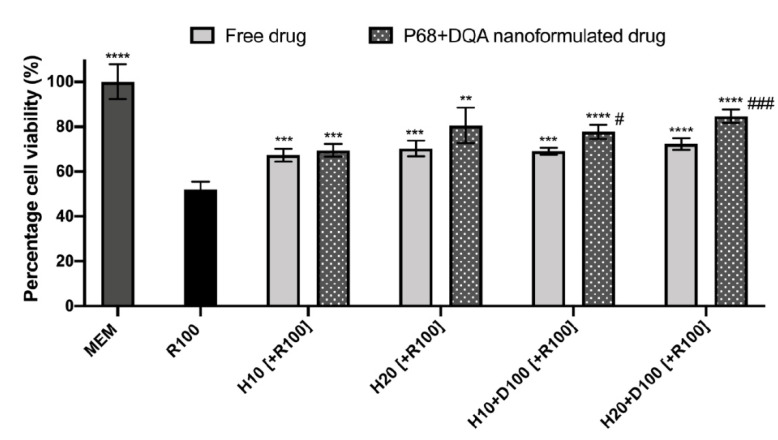
SH-SY5Y MTT assay results for free and P68 + DQA preparations of 10 and 20 µM HT and combined HT and DF0 (10 or 20 µM HT + 50 or 100 µM DFO, respectively) following passage across the hCMEC/D3-SH-SY5Y co-culture Transwell^®^ system. The hCMEC/D3 cells were grown on the insert and the SH-SY5Y cells were located at the bottom of the basolateral compartment. Treatments were added to the apical compartment of the Transwell^®^ system and incubated for 3 h, the SH-SY5Y cells were then incubated with 100 µM rotenone for 24 h. These results were compared to rotenone treatment alone. MEM represents the control condition where cells were only treated with media (mean ± S.D., *n* = 6). * represents significance values of control or pre-treatment conditions compared to rotenone treatment alone (**** *p* < 0.0001, *** *p* < 0.001, ** *p* < 0.01). # represents significance values of nanoformulated drug compared to free drug within the same treatment condition (### *p* < 0.001, # *p* < 0.05).

When assessing the ability of free and P68 + DQA HT and HT + DFO to protect against rotenone induced cytotoxicity following BBB passage, significant differences were observed (F(9, 22.26) = 49.87, *p* < 0.0001) (Figure 7). All free and P68 + DQA HT and HT + DFO pre-treatments resulted in significantly higher cell viability compared to rotenone treatment alone (10 µM HT-free: *p* = 0.0003, P68 + DQA: *p* = 0.0002, 20 µM HT-free: *p* = 0.0001, P68 + DQA: *p* = 0.0023, 10 µM HT + 50 µM DFO-free: *p* = 0.0004, P68 + DQA: *p* < 0.0001 and 20 µM HT + 100 µM DFO-free & P68 + DQA: *p* < 0.0001), however, only P68 + DQA 20 µM HT and P68 + DQA 20 µM HT + 100 µM DFO maintained cell viability at 80% of control (Figure 7). The P68 + DQA nanoformulations of both concentrations of combined HT and DFO resulted in significantly higher cell viability compared to the corresponding free drug conditions (10 µM HT + 50 µM DFO: 8.7%, *p* = 0.0125 and 20 µM HT + 100 µM DFO: 12.3%, *p* = 0.0006, respectively) (Figure 7).

**Figure 8:**
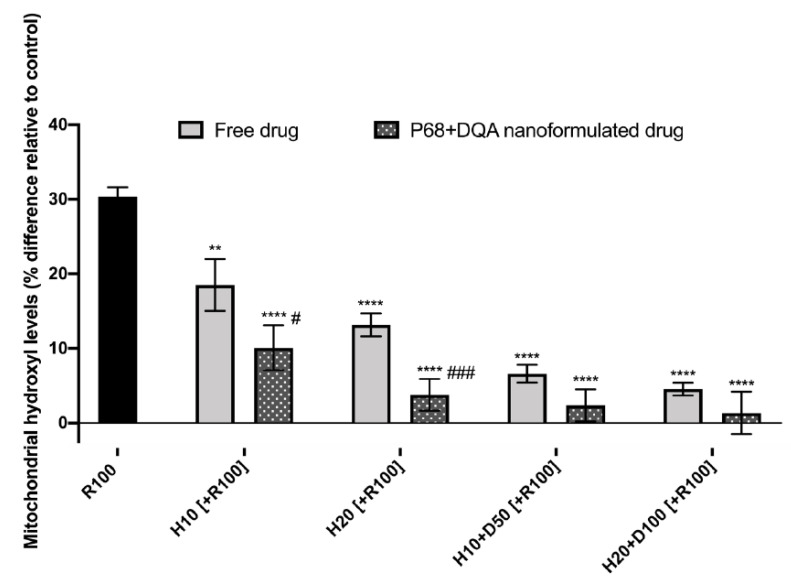
SH-SY5Y mitochondrial hydroxyl assay results for free and P68 + DQA preparations of 10 and 20 µM HT and combined HT and DF0 (10 or 20 µM HT + 50 or 100 µM DFO, respectively) following passage across the hCMEC/D3-SH-SY5Y co-culture Transwell^®^ system. The hCMEC/D3 cells were grown on the insert and the SH-SY5Y cells were located at the bottom of the basolateral compartment. Treatments were added to the apical compartment of the Transwell^®^ system and incubated for 3 h, the SH-SY5Y cells were then incubated with 100 µM rotenone for 24 h. These results were compared to rotenone treatment alone. Mitochondrial hydroxyl levels are expressed as the percentage of hydroxyl identified in control cells (SH-SY5Y cells treated with MEM media only, for 24 h). (mean ± S.D., *n* = 6). * represents significance values of control or pre-treatment conditions compared to rotenone treatment alone (**** *p* < 0.0001, ** *p* < 0.01). # represents significance values of nanoformulated drug compared to free drug within the same treatment condition (### *p* < 0.001, # *p* < 0.05).

Mitochondrial hydroxyl levels were also assessed using the Transwell^®^ model to evaluate the ability of the free and nanoformulated treatments to protect against rotenone induced oxidative stress. Significant differences were observed when using the mitochondrial hydroxyl assay to assess the ability of free and P68 + DQA HT and HT + DFO to protect against rotenone induced oxidative stress in the Transwell^®^ model (F(8, 28.41 = 107.9, *p* < 0.0001) (Figure 8). Both 10 and 20 µM P68 + DQA HT conditions resulted in significantly lower levels of hydroxyl compared to the corresponding free drug conditions (*p* = 0.0298 and *p* = 0.0003, respectively) (Figure 8). However, the combination of 10 µM HT and 100 µM DFO in P68 + DQA nanoformulations resulted in the lowest percentage mitochondrial hydroxyl levels relative to control (1.3%) compared to all the other HT and HT + DFO treatments (Figure 8).

## 4. Discussion

There is increasing evidence suggesting that HT is protective in numerous models of PD [2,11,17,18,19,58,59]. Yet, the full therapeutic potential of HT as a disease modifying treatment for PD is unlikely to be reached due to issues such as low bioavailability and stability, lack of targeted delivery, and limited brain delivery [20]. The aim of this study was firstly to assess the ability of the P68 + DQA micellar nanocarriers (developed by Mursaleen et al. [24,25]) to incorporate HT, alone or in combination with DFO, and secondly to assess whether these nanoformulations could protect against reduced cell viability and increased oxidative stress induced by a rotenone model of PD in a hCMEC/D3-SH-SY5Y Transwell^®^ co-culture system.

HT, alone or combined with DFO, was successfully incorporated into P68 + DQA nanocarriers with high loading efficiency (Table 1). This is consistent with the encapsulation efficiencies of these nanocarriers with other antioxidants [24,25]. The HT and HT + DFO P68 + DQA nanocarriers exhibited consistent particle sizes (polydispersity indices < 0.24), each below 170 nm (Table 1), suggesting that these formulations should be of dimensions sufficient to cross the BBB based on previous reports [23,60,61]. The mean surface charges of the HT and HT + DFO P68 + DQA nanoformulations were similarly neutral (+7.43 and +9.87 mV, respectively) (Table 1). These relatively neutral surface charges suggest that these HT P68 + DQA nanocarriers, with and without the combination of DFO, should be able to access the brain without causing toxicity to the BBB [23,28,30,31,62,63].

XRD studies revealed the crystalline nature of free HT and DFO. This was suppressed by formulation into P68 + DQA nanocarriers (Figure 1). This amorphous transformation is of benefit to these formulations due to the known association with increased solubility and stability [64]. This suggests that these HT and HT + DFO P68 + DQA nanoformulations would be suitable for oral or nasal delivery as they should remain stable once ingested or inhaled and would be more easily absorbed into the blood for systemic or neuronal circulation than free HT and HT + DFO, due to the increased solubility [65]. The decrease in intensity of the HT and DFO peaks, with minimal shifting, in the FTIR spectra for the relevant lyophilized formulations compared to the physical mixtures (Figure 2) indicates the incorporation of each of these drugs into the P68 + DQA nanoformulations, without any conjugation interactions between the chemical groups [22,66].

The concentration ranges selected for HT (10–200 μM) and tested in the FRAP and MTT assays were based on and consistent with previous literature [2,67,68,69]. The FRAP results show a correlation between increased concentration of HT and increased antioxidant capacity (Figure 3). Generally, the P68 + DQA HT nanoformulations exhibited significantly higher antioxidant capacity than the corresponding free HT concentrations (Figure 3). This is likely due to the improved stability of HT when loaded into the P68 + DQA nanocarriers as low stability is a possible disadvantage for polyphenols such as HT due to the extraction process [20]. Ultimately, the 10 and 20 μM concentrations of HT were selected for further evaluation as these were the highest concentrations of both the free drug and P68 + DQA nanoformulations that resulted in no observable cytotoxicity in SH-SY5Y cells after treatment for up to 72 h (Figure 4).

The hCMEC/D3 cell line was used to model the BBB in a Transwell^®^ system based on previous studies [47,48,49,50,51,52]. The different free and nanoformulated HT treatments, alone and in combination with DFO, were tested on this model to assess whether they are likely to enter the brain in vivo and to evaluate the protective effects of these treatments against rotenone induced oxidative stress. Importantly, no significant differences in TEER values were observed following treatment with all concentrations of free and P68 + DQA HT and HT + DFO (Figure 5), suggesting that none of these treatments are likely to cause toxicity to the BBB. The results of this study indicate that HT can pass across the BBB to some extent as supported by previous literature [11,18,19]. However, in every case P68 + DQA nanoformulation increased the percentage of HT reaching the basolateral compartment of the Transwell^®^ model by up to 50% (with 10 µM HT) (Figure 6).

Rotenone was used to model PD in this system as it is a pesticide and insecticide that is commonly used to induce the characteristic features of PD in both in vitro and in vivo models [70]. It is a strong inhibitor of mitochondrial complex 1 and has been linked to the higher incidences of PD in agricultural areas [71,72]. Rotenone inhibits electron transfer from the iron-sulphur clusters in complex I to ubiquinone which blocks oxidative phosphorylation and limits ATP synthesis [73]. Such incomplete electron transfer also results in the excessive formation of ROS and together eventually leads to apoptosis of the affected cells [74,75,76]. Unlike other neurotoxin models of PD, rotenone models have been shown to produce the most PD-like motor symptoms in animals as well as the most histopathological hallmarks of PD, from iron accumulation and oxidative stress to Lewy body pathology [77,78,79,80,81,82].

When using the Transwell^®^ model to evaluate the protective effects of free and P68 + DQA HT and HT + DFO against rotenone induced cytotoxicity and mitochondrial hydroxyl in SH-SY5Y cells following passage across the hCMEC/D3 membrane, the P68 + DQA nanoformulated treatments were superior in every case (Figure 7 and Figure 8). This indicates that the P68 + DQA formulations were able to mostly stay intact until reaching the mitochondria within the SH-SY5Y cells, as it is here where rotenone exerts its effects as a mitochondrial complex 1 inhibitor [72]. The highest concentrations of the P68 + DQA combinations of HT and DFO were the most effective of the treatments at protecting against rotenone induced cytotoxicity and increased mitochondrial hydroxyl, in both cases maintaining cell viability above 80% and hydroxyl at least in line with control levels (Figure 7 and Figure 8). However, there was no significant difference between the 20 µM HT and 20 µM HT + 100 µM DFO pre-treatments. This perhaps relates to the reported iron chelating properties of HT [83], suggesting that there may be little added value in combining HT and DFO, despite the combination treatments being the most effective overall.

Taken together, these results suggest that P68 + DQA HT and HT + DFO nanocarriers have the relevant characteristics to access the brain without producing cytotoxicity and protect against rotenone induced oxidative stress.

## 5. Conclusions

This study demonstrates for the first time the incorporation of HT and HT + DFO into P68 + DQA nanocarriers and successful delivery of these nanocarriers across a BBB model to protect against PD-related oxidative stress. These results highlight the benefit of using micellar nanocarriers to improve the passage of HT across biological membranes and enhance its therapeutic effects. The ability of the P68 + DQA nanocarriers to enhance the protective effects of HT and HT + DFO against rotenone induced oxidative stress warrants further investigation in in vivo models as it suggests that these nanocarriers have potential to become therapeutic agents for PD.

## Figures and Tables

**Table 1:** Hydrodynamic diameter (d), polydispersity index (PDI), surface charge, drug loading (DL) and encapsulation efficiency (EE) of blank and drug-loaded P68 + DQA nanoformulations prepared at 80 °C (mean ± S.D., *n* = 6).

Sample	Contents (mg/mL)		*d* (nm)	PDI	Charge (mV)	DL (%)	EE (%)
P68 + DQA (Blank)	P68:	9	25.52 ± 10.25	0.24 ± 0.04	0.78 ± 0.80		
	DQA:	1					
P68 + DQA: HT	P68:	9	166.28 ± 22.41	0.23 ± 0.03	7.43 ± 0.91	8.63 ± 1.16	94.56 ± 13.87
	DQA:	1					
	HT:	1					
P68 + DQA: HT + DFO	P68:	9	146.26 ± 8.88	0.18 ± 0.07	9.87 ± 1.21	HT:	HT:
	DQA:	1				1.69 ± 0.03	97.17 ± 1.81
	HT:	0.24				DFO:	DFO:
	DFO:	5				27.26 ± 0.30	76.72 ± 1.42

## Data Availability

The data presented in this study are available on request from the corresponding author.

## References

[1] Khalatbary A.R. (2013). Olive oil phenols and neuroprotection. Nutr. Neurosci..

[2] Yu G., Deng A., Tang W., Ma J., Yuan C., Ma J. (2016). Hydroxytyrosol induces phase II detoxifying enzyme expression and effectively protects dopaminergic cells against dopamine-and 6-hydroxydopamine induced cytotoxicity. Neurochem. Int..

[3] Trichopoulou A., Costacou T., Bamia C., Trichopoulos D. (2003). Adherence to a Mediterranean diet and survival in a Greek population. N. Engl. J. Med..

[4] Trichopoulou A. (2004). Traditional Mediterranean diet and longevity in the elderly: A review. Public Health Nutr..

[5] Barzi F., Woodward M., Marfisi R.M., Tavazzi L., Valagussa F., Marchioli R. (2003). Mediterranean diet and all-causes mortality after myocardial infarction: Results from the GISSI-Prevenzione trial. Eur. J. Clin. Nutr..

[6] Granados-Principal S., El-Azem N., Pamplona R., Ramirez-Tortosa C., Pulido-Moran M., Vera-Ramirez L., Quiles J.L., Sanchez-Rovira P., Naudí A., Portero-Otin M. (2014). Hydroxytyrosol ameliorates oxidative stress and mitochondrial dysfunction in doxorubicin-induced cardiotoxicity in rats with breast cancer. Biochem. Pharmacol..

[7] Feart C., Samieri C., Rondeau V., Amieva H., Portet F., Dartigues J.F., Scarmeas N., Barberger-Gateau P. (2009). Adherence to a Mediterranean diet, cognitive decline, and risk of dementia. JAMA.

[8] Hashimoto T., Ibi M., Matsuno K., Nakashima S., Tanigawa T., Yoshikawa T., Yabe-Nishimura C. (2004). An endogenous metabolite of dopamine, 3, 4-dihydroxyphenylethanol, acts as a unique cytoprotective agent against oxidative stress-induced injury. Free Radic. Biol. Med..

[9] Han J.M., Lee Y.J., Lee S.Y., Kim E.M., Moon Y., Kim H.W., Hwang O. (2007). Protective effect of sulforaphane against dopaminergic cell death. J. Pharmacol. Exp. Ther..

[10] González-Correa J.A., Navas M.D., Lopez-Villodres J.A., Trujillo M., Espartero J.L., De La Cruz J.P. (2008). Neuroprotective effect of hydroxytyrosol and hydroxytyrosol acetate in rat brain slices subjected to hypoxia–reoxygenation. Neurosci. Lett..

[11] Wu Y.T., Lin L.C., Tsai T.H. (2009). Measurement of free hydroxytyrosol in microdialysates from blood and brain of anesthetized rats by liquid chromatography with fluorescence detection. J. Chromatogr. A.

[12] Ristagno G., Fumagalli F., Porretta-Serapiglia C., Orrù A., Cassina C., Pesaresi M., Masson S., Villanova L., Merendino A., Villanova A. (2012). Hydroxytyrosol attenuates peripheral neuropathy in streptozotocin-induced diabetes in rats. J. Agric. Food Chem..

[13] Pasban-Aliabadi H., Esmaeili-Mahani S., Sheibani V., Abbasnejad M., Mehdizadeh A., Yaghoobi M.M. (2013). Inhibition of 6-hydroxydopamine-induced PC12 cell apoptosis by olive (*Olea europaea* L.) leaf extract is performed by its main component oleuropein. Rejuvenation Res..

[14] Amic D., Davidovic-Amic D., Beslo D., Rastija V., Lucic B., Trinajstic N. (2007). SAR and QSAR of the antioxidant activity of flavonoids. Curr. Med. Chem..

[15] Sandoval-Acuña C., Ferreira J., Speisky H. (2014). Polyphenols and mitochondria: An update on their increasingly emerging ROS-scavenging independent actions. Arch. Biochem. Biophys..

[16] Lombardo L., Grasso F., Lanciano F., Loria S., Monetti E. (2018). Broad-spectrum health protection of extra virgin olive oil compounds. Studies in Natural Products Chemistry.

[17] Hornedo-Ortega R., Cerezo A.B., Troncoso A.M., Garcia-Parrilla M.C. (2018). Protective effects of hydroxytyrosol against α-synuclein toxicity on PC12 cells and fibril formation. Food Chem. Toxicol..

[18] Schaffer S., Podstawa M., Visioli F., Bogani P., Müller W.E., Eckert G.P. (2007). Hydroxytyrosol-rich olive mill wastewater extract protects brain cells in vitro and ex vivo. J. Agric. Food Chem..

[19] D’Angelo S., Manna C., Migliardi V., Mazzoni O., Morrica P., Capasso G.G., Pontoni G., Galletti P., Zappia V. (2001). Pharmacokinetics and metabolism of hydroxytyrosol, a natural antioxidant from olive oil. Drug Metab. Dispos..

[20] Robles-Almazan M., Pulido-Moran M., Moreno-Fernandez J., Ramirez-Tortosa C., Rodriguez-Garcia C., Quiles J.L., Ramirez-Tortosa M. (2018). Hydroxytyrosol: Bioavailability, toxicity, and clinical applications. Food Res. Int..

[21] Masserini M. (2013). Nanoparticles for Brain Drug Delivery. ISRN Biochem..

[22] Zupancčicč S., Kocbek P., Zariwala M.G., Renshaw D., Gul M.O., Elsaid Z., Taylor K.M.G., Somavarapu S. (2014). Design and development of novel mitochondrial targeted nanocarriers, DQAsomes for curcumin inhalation. Mol. Pharm..

[23] Zhou Y., Peng Z., Seven E.S., Leblanc R.M. (2018). Crossing the blood-brain barrier with nanoparticles. J. Control. Release.

[24] Mursaleen L., Somavarapu S., Zariwala M.G. (2020). Deferoxamine and Curcumin Loaded Nanocarriers Protect Against Rotenone-Induced Neurotoxicity. J. Parkinson’s Dis..

[25] Mursaleen L., Noble B., Chan S.H.Y., Somavarapu S., Zariwala M.G. (2020). N-Acetylcysteine nanocarriers protect against oxidative stress in a cellular model of Parkinson*’*s disease. Antioxidants.

[26] Gaucher G., Dufresne M.H., Sant V.P., Kang N., Maysinger D., Leroux J.C. (2005). Block copolymer micelles: Preparation, characterization and application in drug delivery. J. Control. Release.

[27] Batrakova E.V., Kabanov A.V. (2008). Pluronic block copolymers: Evolution of drug delivery concept from inert nanocarriers to biological response modifiers. J. Control. Release.

[28] Huang X., Li L., Liu T., Hao N., Liu H., Chen D., Tang F. (2011). The shape effect of mesoporous silica nanoparticles on biodistribution, clearance, and biocompatibility in vivo. ACS Nano.

[29] Kataoka K., Harada A., Nagasaki Y. (2012). Block copolymer micelles for drug delivery: Design, characterization and biological significance. Adv. Drug Deliv. Rev..

[30] Wiley D.T., Webster P., Gale A., Davis M.E. (2013). Transcytosis and brain uptake of transferrin-containing nanoparticles by tuning avidity to transferrin receptor. Proc. Natl. Acad. Sci. USA.

[31] Bramini M., Ye D., Hallerbach A., Nic Raghnaill M., Salvati A., Aberg C., Dawson K.A. (2014). Imaging approach to mechanistic study of nanoparticle interactions with the blood–brain barrier. ACS Nano.

[32] Elezaby R.S., Gad H.A., Metwally A.A., Geneidi A.S., Awad G.A. (2017). Self-assembled amphiphilic core-shell nanocarriers in line with the modern strategies for brain delivery. J. Control. Release.

[33] Rakotoarisoa M., Angelova A. (2018). Amphiphilic nanocarrier systems for curcumin delivery in neurodegenerative disorders. Medicines.

[34] Dexter D.T., Wells F.R., Agid F., Agid Y., Lees A.J., Jenner P., Marsden C.D. (1987). Increased nigral iron content in postmortem parkinsonian brain. Lancet.

[35] Gerlach M., Ben-Shachar D., Riederer P., Youdim M.B.H. (1994). Altered brain metabolism of iron as a cause of neurodegenerative diseases?. J. Neurochem..

[36] Griffiths P.D., Dobson B.R., Jones G.R., Clarke D.T. (1999). Iron in the basal ganglia in Parkinson’s disease: An in vitro study using extended X-ray absorption fine structure and cryo-electron microscopy. Brain.

[37] Graham J.M., Paley M.N., Grünewald R.A., Hoggard N., Griffiths P.D. (2000). Brain iron deposition in Parkinson’s disease imaged using the PRIME magnetic resonance sequence. Brain.

[38] Halliwell B. (2001). Role of free radicals in the neurodegenerative diseases. Drugs Aging.

[39] Martin W.R.W., Wieler M., Gee M. (2008). Midbrain iron content in early Parkinson disease A potential biomarker of disease status. Neurology.

[40] Wallis L.I., Paley M.N., Graham J.M., Grünewald R.A., Wignall E.L., Joy H.M., Griffiths P.D. (2008). MRI assessment of basal ganglia iron deposition in Parkinson’s disease. J. Magn. Reson. Imaging.

[41] Rossi M., Ruottinen H., Soimakallio S., Elovaara I., Dastidar P. (2013). Clinical MRI for iron detection in Parkinson’s disease. Clin. Imaging.

[42] Kandola K., Bowman A., Birch-Machin M.A. (2015). Oxidative stress–a key emerging impact factor in health, ageing, lifestyle and aesthetics. Int. J. Cosmet. Sci..

[43] Costa-Mallen P., Gatenby C., Friend S., Maravilla K.R., Hu S.C., Cain K.C., Agarwal P., Anzai Y. (2017). Brain iron concentrations in regions of interest and relation with serum iron levels in Parkinson disease. J. Neurol. Sci..

[44] Weissig V., Lasch J., Erdos G., Meyer H.W., Rowe T.C., Hughes J. (1998). DQAsomes: A novel potential drug and gene delivery system made from Dequalinium. Pharm. Res..

[45] Anon A.O.A.C. (2012). Standard method performance requirements for in vitro determination of total antioxidant activity in foods, beverages, food ingredients, and dietary supplements. J. AOAC Int..

[46] Yusof H.I., Owusu-Apenten R., Nigam P.S. (2018). Determination of iron (III) reducing antioxidant capacity for manuka honey and comparison with ABTS and other methods. J. Adv. Biol. Biotech..

[47] Weksler B.B., Subileau E.A., Perriere N., Charneau P., Holloway K., Leveque M., Tricoire-Leignel H., Nicotra A., Bourdoulous S., Turowski P. (2005). Blood-brain barrier-specific properties of a human adult brain endothelial cell line. FASEB J..

[48] Cristante E., McArthur S., Mauro C., Maggioli E., Romero I.A., Wylezinska-Arridge M., Couraud P.O., Lopez-Tremoleda J., Christian H.C., Weksler B.B. (2013). Identification of an essential endogenous regulator of blood–brain barrier integrity, and its pathological and therapeutic implications. Proc. Natl. Acad. Sci. USA.

[49] Weksler B., Romero I.A., Couraud P.O. (2013). The hCMEC/D3 cell line as a model of the human blood brain barrier. Fluids Barriers CNS.

[50] Maggioli E., McArthur S., Mauro C., Kieswich J., Kusters D.H.M., Reutelingsperger C.P.M., Yaqoob M., Solito E. (2016). Estrogen protects the blood–brain barrier from inflammation-induced disruption and increased lymphocyte trafficking. Brain Behav. Immun..

[51] Paradis A., Leblanc D., Dumais N. (2016). Optimization of an in vitro human blood–brain barrier model: Application to blood monocyte transmigration assays. MethodsX.

[52] Hoyles L., Snelling T., Umlai U.K., Nicholson J.K., Carding S.R., Glen R.C., McArthur S. (2018). Microbiome–host systems interactions: Protective effects of propionate upon the blood–brain barrier. Microbiome.

[53] Gonzalez-Carter D., Goode A.E., Kiryushko D., Masuda S., Hu S., Lopes-Rodrigues R., Dexter D.T., Shaffer M.S., Porter A.E. (2019). Quantification of blood–brain barrier transport and neuronal toxicity of unlabelled multiwalled carbon nanotubes as a function of surface charge. Nanoscale.

[54] Burkhart A., Skjørringe T., Johnsen K.B., Siupka P., Thomsen L.B., Nielsen M.S., Thomsen L.L., Moos T. (2016). Expression of iron-related proteins at the neurovascular unit supports reduction and reoxidation of iron for transport through the blood-brain barrier. Mol. Neurobiol..

[55] Molino Y., Jabès F., Lacassagne E., Gaudin N., Khrestchatisky M. (2014). Setting-up an in vitro model of rat blood-brain barrier (BBB): A focus on BBB impermeability and receptor-mediated transport. JoVE.

[56] Bressler J., Clark K., O’Driscoll C. (2013). Assessing Blood–Brain Barrier Function Using In Vitro Assays. Cell-Cell Interactions.

[57] Åberg C. (2016). Quantitative analysis of nanoparticle transport through in vitro blood-brain barrier models. Tissue Barriers.

[58] Sarbishegi M., Gorgich E.A.C., Khajavi O., Komeili G., Salimi S. (2018). The neuroprotective effects of hydro-alcoholic extract of olive (Olea europaea L.) leaf on rotenone-induced Parkinson’s disease in rat. Metab. Brain Dis..

[59] Goldstein D.S., Jinsmaa Y., Sullivan P., Holmes C., Kopin I.J., Sharabi Y. (2016). 3, 4-Dihydroxyphenylethanol (hydroxytyrosol) mitigates the increase in spontaneous oxidation of dopamine during monoamine oxidase inhibition in PC12 cells. Neurochem. Res..

[60] Cruz L.J., Stammes M.A., Que I., van Beek E.R., Knol-Blankevoort V.T., Snoeks T.J.A., Chan A., Kaijzel E.L., Löwik C.W.G.M. (2016). Effect of PLGA NP size on efficiency to target traumatic brain injury. J. Control. Release.

[61] Grabrucker A.M., Ruozi B., Belletti D., Pederzoli F., Forni F., Vandelli M.A., Tosi G. (2016). Nanoparticle transport across the blood brain barrier. Tissue Barriers.

[62] Lockman P.R., Koziara J.M., Mumper R.J., Allen D.D. (2004). Nanoparticle surface charges alter blood–brain barrier integrity and permeability. J. Drug Target..

[63] Choi J.J., Wang S., Tung Y.S., Morrison B., Konofagou E.E. (2010). Molecules of various pharmacologically-relevant sizes can cross the ultrasound-induced blood-brain barrier opening in vivo. Ultrasound Med. Biol..

[64] Shi M., Zhang J., Li X., Pan S., Li J., Yang C., Hu H., Qiao M., Chen D., Zhao X. (2018). Mitochondria-targeted delivery of doxorubicin to enhance antitumor activity with HER-2 peptide-mediated multifunctional pH-sensitive DQAsomes. Int. J. Nanomed..

[65] Savjani K.T., Gajjar A.K., Savjani J.K. (2012). Drug solubility: Importance and enhancement techniques. Int. Sch. Res. Not..

[66] Bourassa P., Kanakis C.D., Tarantilis P., Pollissiou M.G., Tajmir-Riahi H.A. (2010). Resveratrol, genistein, and curcumin bind bovine serum albumin. J. Phys. Chem. B.

[67] Yamamuro A., Yoshioka Y., Ogita K., Maeda S. (2006). Involvement of endoplasmic reticulum stress on the cell death induced by 6-hydroxydopamine in human neuroblastoma SH-SY5Y cells. Neurochem. Res..

[68] de las Hazas M.C.L., Godinho-Pereira J., Macià A., Almeida A.F., Ventura M.R., Motilva M.J., Santos C.N. (2018). Brain uptake of hydroxytyrosol and its main circulating metabolites: Protective potential in neuronal cells. J. Funct. Foods.

[69] Funakohi-Tago M., Sakata T., Fujiwara S., Sakakura A., Sugai T., Tago K., Tamura H. (2018). Hydroxytyrosol butyrate inhibits 6-OHDA-induced apoptosis through activation of the Nrf2/HO-1 axis in SH-SY5Y cells. Eur. J. Pharmacol..

[70] Xicoy H., Wieringa B., Martens G.J. (2017). The SH-SY5Y cell line in Parkinson’s disease research: A systematic review. Mol. Neurodegener..

[71] Gorell J.M., Johnson C.C., Rybicki B.A., Peterson E.L., Richardson R.J. (1998). The risk of Parkinson’s disease with exposure to pesticides, farming, well water, and rural living. Neurology.

[72] Tanner C.M., Kamel F., Ross G.W., Hoppin J.A., Goldman S.M., Korell M., Marras C., Bhudhikanok G.S., Kasten M., Chade A.R. (2011). Rotenone, paraquat, and Parkinson’s disease. Environ. Health Perspect..

[73] Palmer G., Horgan D.J., Tisdale H., Singer T.P., Beinert H. (1968). Studies on the respiratory chain-linked reduced nicotinamide adenine dinucleotide dehydrogenase: XIV. Location of the sites of inhibition of rotenone, barbiturates, and piericidin by means of electron paramagnetic resonance spectroscopy. J. Biol. Chem..

[74] Li N., Ragheb K., Lawler G., Sturgis J., Rajwa B., Melendez J.A., Robinson J.P. (2003). Mitochondrial complex I inhibitor rotenone induces apoptosis through enhancing mitochondrial reactive oxygen species production. J. Biol. Chem..

[75] Fato R., Bergamini C., Bortolus M., Maniero A.L., Leoni S., Ohnishi T., Lenaz G. (2009). Differential effects of mitochondrial Complex I inhibitors on production of reactive oxygen species. Biochim. Biophys. Acta (BBA)-Bioenergy.

[76] Heinz S., Freyberger A., Lawrenz B., Schladt L., Schmuck G., Ellinger-Ziegelbauer H. (2017). Mechanistic investigations of the mitochondrial complex I inhibitor rotenone in the context of pharmacological and safety evaluation. Sci. Rep..

[77] Sherer T.B., Betarbet R., Kim J.H., Greenamyre J.T. (2003). Selective microglial activation in the rat rotenone model of Parkinson*’*s disease. Neurosci. Lett..

[78] Betarbet R., Sherer T.B., MacKenzie G., Garcia-Osuna M., Panov A.V., Greenamyre J.T. (2000). Chronic systemic pesticide exposure reproduces features of Parkinson*’*s disease. Nat. Neurosci..

[79] Betarbet R., Canet-Aviles R.M., Sherer T.B., Mastroberardino P.G., McLendon C., Kim J.H., Greenamyre J.T. (2006). Intersecting pathways to neurodegeneration in Parkinson’s disease: Effects of the pesticide rotenone on DJ-1, α-synuclein, and the ubiquitin–proteasome system. Neurobiol. Dis..

[80] Chinta S.J., Lieu C.A., DeMaria M., Laberge R.M., Campisi J., Andersen J.K. (2013). Environmental stress, ageing and glial cell senescence: A novel mechanistic link to Parkinson’s disease?. J. Intern. Med..

[81] Mouhape C., Costa G., Ferreira M., Abin-Carriquiry J.A., Dajas F., Prunell G. (2019). Nicotine-induced neuroprotection in rotenone in vivo and in vitro models of Parkinson’s disease: Evidences for the involvement of the labile iron pool level as the underlying mechanism. Neurotox. Res..

[82] Siracusa R., Scuto M., Fusco R., Trovato A., Ontario M.L., Crea R., Di Paola R., Cuzzocrea S., Calabrese V. (2020). Anti-inflammatory and Anti-oxidant Activity of Hidrox^®^ in Rotenone-Induced Parkinson’s Disease in Mice. Antioxidants.

[83] Kitsati N., Mantzaris M.D., Galaris D. (2016). Hydroxytyrosol inhibits hydrogen peroxide-induced apoptotic signaling via labile iron chelation. Redox Biol..

